# Bis(4-phenyl-2-sulfanyl­idene-2,3-di­hydro-1,3-thia­zol-3-ido-κ^2^ *S* ^2^,*N*)(4-phenyl-1,3-thia­zole-2-thiol­ato-κ*S* ^2^)bis­muth

**DOI:** 10.1107/S241431462000067X

**Published:** 2020-01-28

**Authors:** Hans-Georg Stammler, Muhammad Imran

**Affiliations:** a Department of Chemistry, University of Bielefeld, PO Box 100131, 33501 Bielefeld, Germany; b Institute of Chemistry, University of the Punjab, Lahore, Pakistan; Vienna University of Technology, Austria

**Keywords:** crystal structure, bis­muth, monomeric complex, N,S-donor ligand

## Abstract

The title compound, [Bi(C_9_H_6_NS_2_)_3_], was prepared by reacting BiCl_3_ and 2-mercapto-4-phenyl­thia­zole (*L*H) at room temperature in a stoichiometric ratio of 1:4. The mol­ecular structure reveals a slightly distorted square-pyramidal environment around the Bi^III^ atom. Two of the three monoanionic ligands *L* ^−^ coordinate in an *N*,*S*-bidentate mode, while one shows a monodentate mode through an S atom. There are no significant inter­molecular inter­actions present in the crystal.

## Abstract 2

The Bi^III^ atom is coordinated to three S and two N atoms of three different ligands and exhibits a square-pyramidal coordination environment.

## Graphical abstract

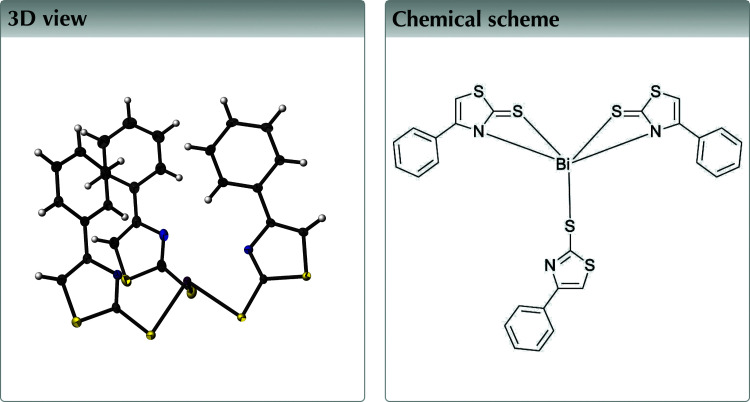

## Structure description

For general background on this type of bis­muth chemistry with S- or (N,S)-donor ligands, see: Diemer *et al.* (1995 ▸); Stavila *et al.* (2006 ▸); Briand *et al.* (2000 ▸). The coordination chemistry of bis­muth with thio­urea or thio­semicarbazide ligands has been studied in detail (Battaglia & Corradi, 1981 ▸,1983 ▸; Battaglia *et al.*, 1992 ▸). While thio­urea ligands have been found to be S-donor ligands only, thio­semicarbazide shows an (N,S)-coordination mode. Recently, we have reported the coordination modes of three heterocyclic ligands derived from 3-mercapto-4-methyl-1,2,4-triazole (*L*1H), 2-mercapto-benzimidazole (*L*2H) and 2-mercapto-4-methyl­thia­zole (*L*3H), respectively, towards bis­muth(III). In the corresponding three bis­muth complexes [Bi(*L*1)_4_(Cl)_2_]Cl, [Bi(*L*2)_4_Cl_2_]^+^[Bi(*L*2)_2_Cl_4_]^−^ and [Bi(*L*3)_2_Cl_2_(μ-Cl)]_2_ (Imran *et al.*, 2013 ▸, 2014 ▸), all these ligands coordinate solely *via* their S-donor atoms despite a possible (N,S) coordination.

**Figure 1:**
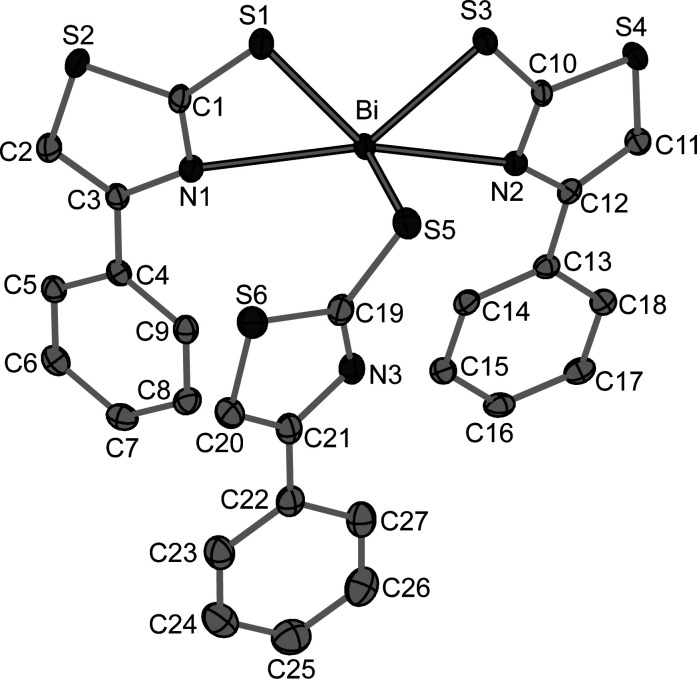
Mol­ecular structure of the title compound, with anisotropic displacement ellipsoids shown at the 50% probability level. Hydrogen atoms are omitted for clarity.

In the title compound, the deprotonated ligand *L* (*L*H is 2-mercapto-4-phenyl thia­zole) exhibits both monodentate S- and bidentate (N,S)-coordination modes (Fig. 1 ▸). Two ligands coordinate in a bidentate fashion (*via* N1, S1, and *via* N2, S3) while the third one exhibits a monodentate mode *via* the S5 donor atom, resulting in a slightly distorted square-pyramidal coordination environment. The Bi—N and Bi—S bonds differ in lengths with the Bi—S bonds shorter by ≃ 0.2 Å (Table 1 ▸) but the index parameter (Addison *et al.*, 1984 ▸) of *τ*_5_ = 0 indicates an ideal value for a square-pyramidal coordination (ideal value for trigonal–bipyramidal coordination is *τ*_5_ = 1).

**Figure 2:**
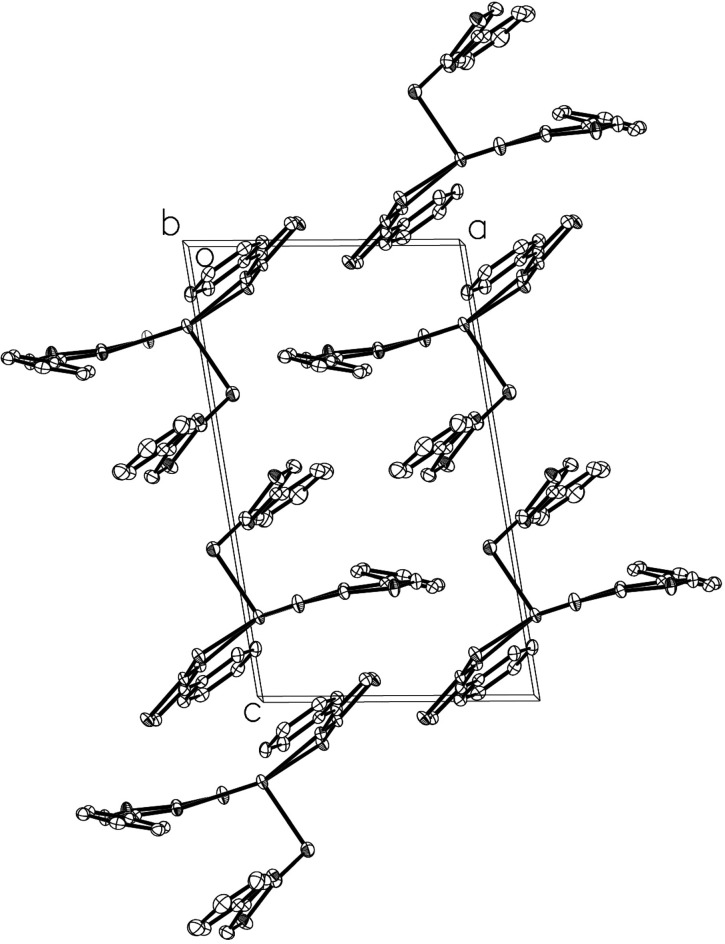
A packing plot of the title compound in a view along the *b* axis.

In the crystal packing (Fig. 2 ▸), no significant inter­molecular inter­actions are found, except a short S⋯S contact between S2 and S5(*x* + 1, *y*, *z*) with a distance of 3.473 (1) Å.

## Synthesis and crystallization

The title compound was prepared by reacting BiCl_3_ (1 mmol, 0.315 g) and 2-mercapto-4-phenyl thia­zole (LH) (4 mmol, 0.773 g) in THF at room temperature. After stirring for 4 h, the resulting yellow solution was concentrated, yielding a yellow solid that was separated by deca­ntation and washed with small amounts of THF followed by diethyl ether. The solid was dried and recrystallized from a mixture of THF/pentane (ratio *v*:*v* = 1:3). Yellow to orange crystals suitable for X-ray diffraction were obtained by slow evaporation of the THF solution of the complex. Yield 76%; m.p. 507 K. ^1^H NMR (CDCl_3_): δ 7.58–7.60 (*dd*, 2H, C2H, C6H), 7.42–7.49 (*m*, 3H, C3H—C5H), 6.78, CH-thia­zole ring); ^13^C NMR (CDCl_3_): δ 188.5 (C9), 142.5 (C8), 129.9 (C2,6), 129.4 (C3,5), 128.1 (C4), 125.9 (C1), 108.9 (C7).

## Refinement

Crystal data, data collection and refinement details are summarized in Table 2 ▸.

## Supplementary Material

Crystal structure: contains datablock(s) I. DOI: 10.1107/S241431462000067X/wm4119sup1.cif

Structure factors: contains datablock(s) I. DOI: 10.1107/S241431462000067X/wm4119Isup2.hkl

Supporting information file. DOI: 10.1107/S241431462000067X/wm4119Isup4.cdx

CCDC reference: 1979022

Additional supporting information:  crystallographic information; 3D view; checkCIF report

## Figures and Tables

**Table 1:** Selected geometric parameters (Å, °)

Bi1—S1	2.6078 (5)	Bi1—N1	2.7970 (17)
Bi1—S3	2.5938 (5)	Bi1—N2	2.7342 (17)
Bi1—S5	2.5550 (6)		
			
S1—Bi1—N1	59.71 (4)	S5—Bi1—S1	94.838 (18)
S1—Bi1—N2	146.17 (4)	S5—Bi1—S3	87.489 (17)
S3—Bi1—S1	88.679 (16)	S5—Bi1—N1	105.90 (4)
S3—Bi1—N1	146.06 (4)	S5—Bi1—N2	96.58 (4)
S3—Bi1—N2	60.22 (4)	N2—Bi1—N1	144.35 (5)

**Table 2:** Experimental details

Crystal data	
Chemical formula	[Bi(C_9_H_6_NS_2_)_3_]
*M*_r_	785.78
Crystal system, space group	Triclinic, *P* 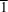
Temperature (K)	100
*a*, *b*, *c* (Å)	9.19758 (16), 10.8904 (2), 14.6041 (2)
α, β, γ (°)	82.0966 (15), 78.5197 (14), 70.9346 (17)
*V* (Å^3^)	1350.77 (4)
*Z*	2
Radiation type	Cu *K*α
μ (mm^−1^)	17.34
Crystal size (mm)	0.12 × 0.06 × 0.03

Data collection	
Diffractometer	Agilent SuperNova, Dual, Cu at zero, Atlas
Absorption correction	Gaussian (*CrysAlis PRO*; Agilent, 2013 ▸)
*T*_min_, *T*_max_	0.085, 0.532
No. of measured, independent and observed [*I* > 2σ(*I*)] reflections	25693, 5325, 5322
*R*_int_	0.021
(sin θ/λ)_max_ (Å^−1^)	0.617

Refinement	
*R*[*F* ^2^ > 2σ(*F* ^2^)], *wR*(*F* ^2^), *S*	0.014, 0.036, 1.11
No. of reflections	5325
No. of parameters	406
H-atom treatment	All H-atom parameters refined
Δρ_max_, Δρ_min_ (e Å^−3^)	0.49, −0.62

Computer programs: *CrysAlis PRO* (Agilent, 2013 ▸), *SHELXS97* (Sheldrick, 2008 ▸) and *OLEX2* (Dolomanov *et al.*, 2009 ▸).

## full crystallographic data

### Crystal data

**Table:**

[Bi(C_9_H_6_NS_2_)_3_]	*Z* = 2
*M**_r_* = 785.78	*F*(000) = 760
Triclinic, *P*1	*D*_x_ = 1.932 Mg m^−^^3^
*a* = 9.19758 (16) Å	Cu *K*α radiation, λ = 1.5418 Å
*b* = 10.8904 (2) Å	Cell parameters from 24519 reflections
*c* = 14.6041 (2) Å	θ = 4.3–76.1°
α = 82.0966 (15)°	µ = 17.34 mm^−^^1^
β = 78.5197 (14)°	*T* = 100 K
γ = 70.9346 (17)°	Needle, orange
*V* = 1350.77 (4) Å^3^	0.12 × 0.06 × 0.03 mm

### Data collection

**Table:**

Agilent SuperNova, Dual, Cu at zero, Atlas diffractometer	5325 independent reflections
Radiation source: SuperNova (Cu) X-ray Source	5322 reflections with *I* > 2σ(*I*)
Mirror monochromator	*R*_int_ = 0.021
Detector resolution: 5.3114 pixels mm^-1^	θ_max_ = 72.0°, θ_min_ = 3.1°
ω scans	*h* = −11→11
Absorption correction: gaussian (*CrysAlis PRO*; Agilent, 2013)	*k* = −12→13
*T*_min_ = 0.085, *T*_max_ = 0.532	*l* = −18→18
25693 measured reflections	

### Refinement

**Table:**

Refinement on *F*^2^	Primary atom site location: structure-invariant direct methods
Least-squares matrix: full	Hydrogen site location: difference Fourier map
*R*[*F*^2^ > 2σ(*F*^2^)] = 0.014	All H-atom parameters refined
*wR*(*F*^2^) = 0.036	*w* = 1/[σ^2^(*F*_o_^2^) + (0.0178*P*)^2^ + 1.2762*P*] where *P* = (*F*_o_^2^ + 2*F*_c_^2^)/3
*S* = 1.11	(Δ/σ)_max_ = 0.006
5325 reflections	Δρ_max_ = 0.49 e Å^−^^3^
406 parameters	Δρ_min_ = −0.62 e Å^−^^3^
0 restraints	

### Special details

**Table:**

Geometry. All esds (except the esd in the dihedral angle between two l.s. planes) are estimated using the full covariance matrix. The cell esds are taken into account individually in the estimation of esds in distances, angles and torsion angles; correlations between esds in cell parameters are only used when they are defined by crystal symmetry. An approximate (isotropic) treatment of cell esds is used for estimating esds involving l.s. planes.

### Fractional atomic coordinates and isotropic or equivalent isotropic displacement parameters (Å^2^)

**Table:**

	*x*	*y*	*z*	*U*_iso_*/*U*_eq_	
Bi1	0.04854 (2)	0.79732 (2)	0.82325 (2)	0.01547 (3)	
S1	0.20152 (6)	0.96640 (5)	0.79684 (4)	0.02075 (11)	
S2	0.55560 (6)	0.85161 (5)	0.76213 (4)	0.02079 (11)	
S3	−0.18891 (6)	0.96840 (5)	0.90975 (3)	0.01687 (10)	
S4	−0.41485 (6)	0.85142 (5)	1.04776 (3)	0.01725 (10)	
S5	−0.07429 (6)	0.87655 (6)	0.67505 (4)	0.02366 (11)	
S6	0.19538 (7)	0.79159 (6)	0.51708 (4)	0.02644 (12)	
N1	0.3712 (2)	0.71876 (17)	0.76281 (12)	0.0160 (3)	
N2	−0.1935 (2)	0.72176 (17)	0.92834 (12)	0.0144 (3)	
N3	0.0621 (2)	0.6322 (2)	0.61607 (12)	0.0203 (4)	
C1	0.3717 (2)	0.8368 (2)	0.77210 (14)	0.0166 (4)	
C2	0.6356 (3)	0.6878 (2)	0.74564 (16)	0.0199 (4)	
H2	0.741 (4)	0.655 (3)	0.734 (2)	0.039 (9)*	
C3	0.5213 (2)	0.6322 (2)	0.74932 (13)	0.0151 (4)	
C4	0.5433 (2)	0.4935 (2)	0.74129 (13)	0.0153 (4)	
C5	0.6842 (2)	0.3977 (2)	0.75534 (14)	0.0172 (4)	
H5	0.765 (3)	0.424 (3)	0.7721 (18)	0.021 (6)*	
C6	0.7044 (3)	0.2675 (2)	0.74796 (15)	0.0195 (4)	
H6	0.801 (3)	0.202 (3)	0.759 (2)	0.025 (7)*	
C7	0.5851 (3)	0.2296 (2)	0.72688 (14)	0.0198 (4)	
H7	0.602 (3)	0.139 (3)	0.720 (2)	0.029 (7)*	
C8	0.4447 (3)	0.3232 (2)	0.71348 (14)	0.0189 (4)	
H8	0.364 (4)	0.297 (3)	0.702 (2)	0.029 (7)*	
C9	0.4235 (2)	0.4543 (2)	0.72059 (14)	0.0169 (4)	
H9	0.328 (3)	0.515 (3)	0.7120 (17)	0.014 (6)*	
C10	−0.2588 (2)	0.8389 (2)	0.95742 (14)	0.0143 (4)	
C11	−0.3889 (2)	0.6881 (2)	1.04507 (15)	0.0165 (4)	
H11	−0.451 (3)	0.648 (3)	1.087 (2)	0.026 (7)*	
C12	−0.2661 (2)	0.6341 (2)	0.97873 (14)	0.0145 (4)	
C13	−0.2010 (2)	0.4953 (2)	0.95995 (14)	0.0149 (4)	
C14	−0.0570 (2)	0.4502 (2)	0.90157 (15)	0.0179 (4)	
H14	−0.004 (3)	0.506 (3)	0.8737 (18)	0.020 (6)*	
C15	0.0088 (3)	0.3187 (2)	0.88689 (16)	0.0198 (4)	
H15	0.103 (4)	0.290 (3)	0.850 (2)	0.029 (7)*	
C16	−0.0690 (3)	0.2307 (2)	0.92946 (15)	0.0193 (4)	
H16	−0.025 (3)	0.141 (3)	0.9200 (18)	0.020 (6)*	
C17	−0.2140 (3)	0.2748 (2)	0.98625 (15)	0.0193 (4)	
H17	−0.269 (3)	0.214 (3)	1.0171 (18)	0.016 (6)*	
C18	−0.2798 (2)	0.4065 (2)	1.00132 (15)	0.0174 (4)	
H18	−0.377 (3)	0.433 (3)	1.0409 (18)	0.018 (6)*	
C19	0.0574 (3)	0.7542 (2)	0.60642 (15)	0.0223 (5)	
C20	0.2623 (3)	0.6324 (2)	0.49126 (16)	0.0239 (5)	
H20	0.344 (4)	0.605 (3)	0.440 (2)	0.032 (8)*	
C21	0.1787 (3)	0.5610 (2)	0.55003 (14)	0.0201 (4)	
C22	0.2029 (3)	0.4211 (2)	0.54815 (15)	0.0208 (4)	
C23	0.3385 (3)	0.3415 (3)	0.49685 (16)	0.0257 (5)	
H23	0.414 (3)	0.379 (3)	0.4648 (19)	0.024 (7)*	
C24	0.3624 (3)	0.2088 (3)	0.49678 (18)	0.0317 (5)	
H24	0.455 (4)	0.155 (3)	0.460 (2)	0.038 (8)*	
C25	0.2526 (3)	0.1534 (3)	0.54776 (19)	0.0335 (6)	
H25	0.272 (4)	0.058 (3)	0.548 (2)	0.040 (9)*	
C26	0.1176 (3)	0.2311 (3)	0.59860 (19)	0.0314 (5)	
H26	0.042 (4)	0.191 (3)	0.632 (2)	0.039 (9)*	
C27	0.0928 (3)	0.3639 (3)	0.59854 (17)	0.0255 (5)	
H27	0.008 (4)	0.415 (3)	0.634 (2)	0.040 (9)*	

### Atomic displacement parameters (Å^2^)

**Table:**

	*U*^11^	*U*^22^	*U*^33^	*U*^12^	*U*^13^	*U*^23^
Bi1	0.01146 (4)	0.01291 (5)	0.02127 (5)	−0.00430 (3)	0.00139 (3)	−0.00338 (3)
S1	0.0142 (2)	0.0136 (2)	0.0332 (3)	−0.00434 (19)	−0.0004 (2)	−0.0026 (2)
S2	0.0136 (2)	0.0185 (2)	0.0324 (3)	−0.00687 (19)	−0.0033 (2)	−0.0052 (2)
S3	0.0153 (2)	0.0113 (2)	0.0222 (2)	−0.00482 (18)	0.00260 (18)	−0.00209 (18)
S4	0.0160 (2)	0.0128 (2)	0.0199 (2)	−0.00403 (18)	0.00428 (18)	−0.00268 (18)
S5	0.0182 (2)	0.0281 (3)	0.0238 (3)	−0.0052 (2)	−0.0023 (2)	−0.0057 (2)
S6	0.0263 (3)	0.0305 (3)	0.0244 (3)	−0.0150 (2)	0.0043 (2)	−0.0055 (2)
N1	0.0140 (8)	0.0163 (9)	0.0178 (8)	−0.0054 (7)	−0.0020 (6)	−0.0010 (7)
N2	0.0136 (8)	0.0131 (8)	0.0164 (8)	−0.0042 (7)	−0.0020 (6)	−0.0016 (6)
N3	0.0161 (9)	0.0288 (10)	0.0161 (8)	−0.0074 (8)	−0.0020 (7)	−0.0019 (7)
C1	0.0128 (9)	0.0198 (10)	0.0179 (9)	−0.0074 (8)	−0.0004 (7)	−0.0009 (8)
C2	0.0144 (10)	0.0188 (11)	0.0260 (11)	−0.0041 (8)	−0.0029 (8)	−0.0036 (8)
C3	0.0129 (9)	0.0185 (10)	0.0134 (9)	−0.0042 (8)	−0.0015 (7)	−0.0017 (7)
C4	0.0157 (10)	0.0169 (10)	0.0122 (9)	−0.0048 (8)	−0.0001 (7)	−0.0006 (7)
C5	0.0151 (10)	0.0210 (11)	0.0147 (9)	−0.0049 (8)	−0.0016 (7)	−0.0023 (8)
C6	0.0195 (11)	0.0187 (11)	0.0169 (10)	−0.0030 (9)	−0.0013 (8)	0.0000 (8)
C7	0.0259 (11)	0.0169 (11)	0.0158 (9)	−0.0074 (9)	−0.0003 (8)	−0.0015 (8)
C8	0.0206 (10)	0.0219 (11)	0.0165 (9)	−0.0101 (9)	−0.0020 (8)	−0.0017 (8)
C9	0.0153 (10)	0.0196 (11)	0.0151 (9)	−0.0054 (8)	−0.0019 (7)	0.0000 (8)
C10	0.0106 (9)	0.0150 (10)	0.0163 (9)	−0.0043 (7)	0.0006 (7)	−0.0016 (7)
C11	0.0171 (10)	0.0134 (10)	0.0186 (10)	−0.0060 (8)	−0.0009 (8)	0.0002 (8)
C12	0.0145 (9)	0.0142 (10)	0.0161 (9)	−0.0064 (8)	−0.0031 (7)	0.0002 (7)
C13	0.0159 (10)	0.0141 (10)	0.0155 (9)	−0.0043 (8)	−0.0052 (7)	−0.0011 (7)
C14	0.0164 (10)	0.0158 (10)	0.0235 (10)	−0.0068 (8)	−0.0035 (8)	−0.0033 (8)
C15	0.0152 (10)	0.0185 (11)	0.0254 (11)	−0.0022 (8)	−0.0048 (8)	−0.0065 (8)
C16	0.0216 (11)	0.0121 (10)	0.0249 (11)	−0.0020 (8)	−0.0092 (8)	−0.0037 (8)
C17	0.0245 (11)	0.0164 (10)	0.0201 (10)	−0.0092 (9)	−0.0072 (8)	0.0009 (8)
C18	0.0174 (10)	0.0171 (10)	0.0181 (10)	−0.0060 (8)	−0.0023 (8)	−0.0019 (8)
C19	0.0172 (10)	0.0311 (13)	0.0202 (10)	−0.0092 (9)	−0.0029 (8)	−0.0030 (9)
C20	0.0225 (11)	0.0300 (13)	0.0190 (10)	−0.0093 (10)	0.0006 (9)	−0.0049 (9)
C21	0.0159 (10)	0.0290 (12)	0.0153 (9)	−0.0060 (9)	−0.0038 (8)	−0.0018 (8)
C22	0.0184 (10)	0.0276 (12)	0.0171 (10)	−0.0065 (9)	−0.0063 (8)	−0.0006 (8)
C23	0.0227 (11)	0.0329 (13)	0.0203 (11)	−0.0077 (10)	−0.0024 (9)	−0.0024 (9)
C24	0.0314 (13)	0.0329 (14)	0.0268 (12)	−0.0037 (11)	−0.0037 (10)	−0.0058 (10)
C25	0.0375 (15)	0.0262 (13)	0.0375 (14)	−0.0082 (11)	−0.0112 (11)	−0.0014 (11)
C26	0.0286 (13)	0.0312 (14)	0.0361 (13)	−0.0128 (11)	−0.0073 (11)	0.0037 (11)
C27	0.0208 (11)	0.0295 (13)	0.0250 (11)	−0.0066 (10)	−0.0047 (9)	0.0008 (10)

### Geometric parameters (Å, º)

**Table:**

Bi1—S1	2.6078 (5)	C8—H8	0.93 (3)
Bi1—S3	2.5938 (5)	C8—C9	1.391 (3)
Bi1—S5	2.5550 (6)	C9—H9	0.93 (3)
Bi1—N1	2.7970 (17)	C11—H11	0.92 (3)
Bi1—N2	2.7342 (17)	C11—C12	1.359 (3)
S1—C1	1.744 (2)	C12—C13	1.475 (3)
S2—C1	1.727 (2)	C13—C14	1.401 (3)
S2—C2	1.724 (2)	C13—C18	1.396 (3)
S3—C10	1.741 (2)	C14—H14	0.90 (3)
S4—C10	1.728 (2)	C14—C15	1.389 (3)
S4—C11	1.721 (2)	C15—H15	0.91 (3)
S5—C19	1.754 (2)	C15—C16	1.387 (3)
S6—C19	1.735 (2)	C16—H16	0.95 (3)
S6—C20	1.708 (3)	C16—C17	1.396 (3)
N1—C1	1.313 (3)	C17—H17	0.97 (3)
N1—C3	1.388 (3)	C17—C18	1.392 (3)
N2—C10	1.310 (3)	C18—H18	0.94 (3)
N2—C12	1.388 (3)	C20—H20	0.95 (3)
N3—C19	1.304 (3)	C20—C21	1.370 (3)
N3—C21	1.389 (3)	C21—C22	1.470 (3)
C2—H2	0.91 (3)	C22—C23	1.403 (3)
C2—C3	1.363 (3)	C22—C27	1.394 (3)
C3—C4	1.475 (3)	C23—H23	0.93 (3)
C4—C5	1.405 (3)	C23—C24	1.388 (4)
C4—C9	1.401 (3)	C24—H24	0.96 (3)
C5—H5	0.96 (3)	C24—C25	1.383 (4)
C5—C6	1.385 (3)	C25—H25	0.99 (3)
C6—H6	0.96 (3)	C25—C26	1.389 (4)
C6—C7	1.391 (3)	C26—H26	0.95 (3)
C7—H7	0.97 (3)	C26—C27	1.388 (4)
C7—C8	1.390 (3)	C27—H27	0.91 (3)
			
S1—Bi1—N1	59.71 (4)	N2—C10—S4	114.33 (15)
S1—Bi1—N2	146.17 (4)	S4—C11—H11	120.4 (18)
S3—Bi1—S1	88.679 (16)	C12—C11—S4	110.94 (16)
S3—Bi1—N1	146.06 (4)	C12—C11—H11	128.6 (18)
S3—Bi1—N2	60.22 (4)	N2—C12—C13	118.96 (18)
S5—Bi1—S1	94.838 (18)	C11—C12—N2	114.04 (18)
S5—Bi1—S3	87.489 (17)	C11—C12—C13	126.91 (19)
S5—Bi1—N1	105.90 (4)	C14—C13—C12	120.24 (19)
S5—Bi1—N2	96.58 (4)	C18—C13—C12	120.76 (19)
N2—Bi1—N1	144.35 (5)	C18—C13—C14	118.98 (19)
C1—S1—Bi1	87.45 (7)	C13—C14—H14	120.6 (17)
C2—S2—C1	89.48 (11)	C15—C14—C13	120.7 (2)
C10—S3—Bi1	86.69 (7)	C15—C14—H14	118.7 (17)
C11—S4—C10	89.19 (10)	C14—C15—H15	120.4 (19)
C19—S5—Bi1	95.96 (8)	C16—C15—C14	120.0 (2)
C20—S6—C19	89.33 (12)	C16—C15—H15	119.6 (19)
C1—N1—Bi1	89.15 (12)	C15—C16—H16	120.8 (17)
C1—N1—C3	111.55 (17)	C15—C16—C17	119.8 (2)
C3—N1—Bi1	156.38 (14)	C17—C16—H16	119.4 (17)
C10—N2—Bi1	90.38 (12)	C16—C17—H17	120.7 (15)
C10—N2—C12	111.48 (17)	C18—C17—C16	120.3 (2)
C12—N2—Bi1	155.75 (13)	C18—C17—H17	119.0 (16)
C19—N3—C21	110.92 (19)	C13—C18—H18	121.5 (16)
S2—C1—S1	122.72 (13)	C17—C18—C13	120.2 (2)
N1—C1—S1	123.07 (16)	C17—C18—H18	118.2 (16)
N1—C1—S2	114.17 (16)	S6—C19—S5	120.09 (14)
S2—C2—H2	117 (2)	N3—C19—S5	125.26 (17)
C3—C2—S2	110.56 (16)	N3—C19—S6	114.65 (17)
C3—C2—H2	132 (2)	S6—C20—H20	120.4 (19)
N1—C3—C4	119.18 (18)	C21—C20—S6	110.71 (18)
C2—C3—N1	114.21 (19)	C21—C20—H20	128.8 (19)
C2—C3—C4	126.61 (19)	N3—C21—C22	119.7 (2)
C5—C4—C3	120.68 (19)	C20—C21—N3	114.4 (2)
C9—C4—C3	120.76 (19)	C20—C21—C22	125.9 (2)
C9—C4—C5	118.6 (2)	C23—C22—C21	120.8 (2)
C4—C5—H5	119.0 (16)	C27—C22—C21	120.8 (2)
C6—C5—C4	120.6 (2)	C27—C22—C23	118.4 (2)
C6—C5—H5	120.4 (16)	C22—C23—H23	118.3 (18)
C5—C6—H6	120.1 (17)	C24—C23—C22	120.7 (2)
C5—C6—C7	120.4 (2)	C24—C23—H23	121.0 (18)
C7—C6—H6	119.4 (17)	C23—C24—H24	120 (2)
C6—C7—H7	119.4 (17)	C25—C24—C23	120.2 (2)
C8—C7—C6	119.6 (2)	C25—C24—H24	120 (2)
C8—C7—H7	121.0 (17)	C24—C25—H25	119.3 (19)
C7—C8—H8	119.5 (19)	C24—C25—C26	119.7 (3)
C7—C8—C9	120.3 (2)	C26—C25—H25	121.0 (19)
C9—C8—H8	120.1 (19)	C25—C26—H26	118 (2)
C4—C9—H9	120.8 (16)	C27—C26—C25	120.3 (2)
C8—C9—C4	120.5 (2)	C27—C26—H26	121 (2)
C8—C9—H9	118.7 (16)	C22—C27—H27	118 (2)
S4—C10—S3	123.72 (12)	C26—C27—C22	120.7 (2)
N2—C10—S3	121.93 (15)	C26—C27—H27	121 (2)
